# Pyridinoacridine alkaloids of marine origin: NMR and MS spectral data, synthesis, biosynthesis and biological activity

**DOI:** 10.3762/bjoc.11.183

**Published:** 2015-09-18

**Authors:** Louis P Sandjo, Victor Kuete, Maique W Biavatti

**Affiliations:** 1 Department of Pharmaceutical Sciences, CCS, Universidade Federal de Santa Catarina, Florianopolis 88040-900, SC, Brazil; 2 Department of Biochemistry, Faculty of Sciences, University of Dschang, Cameroon

**Keywords:** biosynthesis, ^13^C NMR shifts, pharmacophores, pyridoacridines, synthesis

## Abstract

This review focuses on pyridoacridine-related metabolites as one biologically interesting group of alkaloids identified from marine sources. They are produced by marine sponges, ascidians and tunicates, and they are structurally comprised of four to eight fused rings including heterocycles. Acridine, acridone, dihydroacridine, and quinolone cores are features regularly found in these alkaloid skeletons. The lack of hydrogen atoms next to quaternary carbon atoms for two or three rings makes the chemical shift assignment a difficult task. In this regard, one of the aims of this review is the compilation of previously reported, pyridoacridine ^13^C NMR data. Observations have been made on the delocalization of electrons and the presence of some functional groups that lead to changes in the chemical shift of some carbon resonances. The lack of mass spectra information for these alkaloids due to the compactness of their structures is further discussed. Moreover, the biosynthetic pathways of some of these metabolites have been shown since they could inspire biomimetic synthesis. The synthesis routes used to prepare members of these marine alkaloids (as well as their analogues), which are synthesized for biological purposes are also discussed. Pyridoacridines were found to have a large spectrum of bioactivity and this review highlights and compares the pharmacophores that are responsible for the observed bioactivity.

## Introduction

In order to improve food production, healthcare and their living space, humans have developed and improved techniques to study and understand the environment. One of such interests is the chemical investigation of marine organisms, which can provide many compounds belonging to a large class of secondary metabolites. It is well known that these chemicals play specific roles for their host with respect to communication [1], sexual attraction [2] and defense [3]. Marine biodiversity has provided around three thousand new chemicals, the most predominant of which are associated with polyketides, peptides, terpenoids, phenolics, polysaccharides and alkaloids [4]. Various bioactivity functions such as anticancer [5–7], phytotoxicity [8–11], antioxidant [12–16], antimicrobial [17–19], analgesic [20–21], hypotensive [22], hypoglycemic [23], antiprotozoal [24] and plant protecting [25–26] effects, have been exhibited by these marine-derived metabolites. Additionally, some of them also inhibited many enzymes including Na^+^/K^+^-ATPase [26], tyrosine kinase [27], phosphatidylinositol-specific phospholipase C [28], topoisomerase II [29], cathepsin L [30], protein tyrosine phosphatase B (PtpB) [31] and serine protease [32].

The focus of this review is on pyridoacridine-related metabolites as one of the many interesting groups of alkaloids produced from marine sources. They are generally produced by marine sponges, ascidians [33] and tunicates [34] and they are structurally comprised of four to eight fused rings including heterocycles [34–39]. Acridine, acridone, dihydroacridine, and quinolone cores [33,40] are features regularly found in these alkaloid skeletons. The high conjugation of their structure induces a strong electron delocalization, leading to yellow, red, blue, or purple pigmentation [33]. They are isolated either as cationic salts [41] or without any charge [33,41]. To date, these marine alkaloids have been documented as topoisomerase II inhibitors [42], antimicrobials [34], cytotoxic [41], antiviral [41], anti-HIV [43] compounds, and can also interact with DNA [41].

## Review

### Chemistry

#### Structure elucidation: ^13^C NMR data

The difficulty of NMR data assignment could be related to the presence of a high number of quaternary carbons in tetra- to octaheterocycles of these alkaloids. For example, up to eleven quaternary carbons may be involved in the fusion of aromatic rings, which makes this assignment a difficult task. An initial solution for structure determination might be to associate a single crystal X-ray structure to the NMR data. Unfortunately, the development of a suitable crystal for the crystallographic analysis is not an easy task since it depends on the purity of the compound as well as the choice of the solvent or mixture of solvents. Thus, the assignment of the chemical shifts of quaternary carbons using NMR techniques (^1^H, ^13^C, HSQC, HMBC) becomes a challenging issue, especially if there are no neighboring hydrogen atoms. Fortunately, NMR techniques such as 1,n-ADEQUATE can be used to solve the problem of carbon assignment but the experimental time is long, the sensitivity is poor, and a substantial amount of sample is required. However, pyridoacridines present some structural features that can be used for comparison in order to determine the structure of similar compounds. For instance, comparison of the ^13^C NMR data (Tables 1–5) of compounds **1–13** (Table 6) revealed the chemical shift of C-1 (δ 131.0) to be downfield when the B ring is aromatic (compounds **1**, and **9**, **11**–**13**) [42–50].

**Table 1:** ^13^C NMR data of pyridoacridine alkaloids **1**–**8**.

	(125 MHz, CDCl_3_) [42]	(125 MHz, CD_3_OD) [44]	(125 MHz, DMSO-*d*_6_) [45]				(125 MHz, DMSO-*d*_6_) [46]	

Position	**1**	**2**	**3**	**4**	**5**	**6**	**7**	**8**

1	131.9	132.7	117.9	117.6	117.9	117.6	116.6	116.7
2	131.9	133.3	134.8	135.0	132.6	134.7	133.2	133.8
3	129.8	131.4	122.6	122.0	123.0	122.4	124.7	125.0
4	122.8	124.9	125.4	125.4	124.4	125.2	127.0	127.3
4a	121.8	123.1	113.9	113.9	116.7	113.8	119.0	118.7
4b	136.9	139.1	149.1	149.2	140.9	148.9	nd	138.6
5	119.0	122.0	105.5	104.9	109.3	105.1	110.6	110.6
6	149.8	150.9	143.6	143.3	151.6	143.3	141.6	140.5
7a	146.5	147.7	127.0	125.9	142.8	126.7	130.0	nd
8	183.3	181.1	137.3	136.6	139.0	137.2	143.9	142.9
9	132.8	144.7	117.4	121.6	113.6	121.8	109.5	110.2
10	152.2	150.2	118.3	116.2	108.4	113.8	117.5	117.1
10a	150.3	150.7	126.6	128.3	134.6	128.3	113.1	118.0
10b	117.8	118.8	120.3	120.3	117.9	120.4	116.4	113.0
11a	145.3	147.1	140.9	141.0	137.1	141.0	136.0	136.0
12	31.7	29.1	64.3	30.3	191.8	28.2	106.2	106.3
13	39.3	40.2	44.0	37.7	–	37.9	131.4	131.4

	acetyl		trifluoroacetyl		–	–	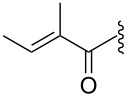	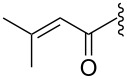

C=O	170.4	–	–	171.4	–	–	169.5	169.6
–	23.3 CH_3_	17.9 *S*CH_3_	–	22.4 CF_3_	–	–	131.2 C	117.6 CH
–	–	–	–	–	–	–	133.0 CH	154.5 C
–	–	–	–	–	–	–	14.0 CH_3_	27.4 CH_3_
–	–	–	–	–	–	–	12.3 CH_3_	19.8 CH_3_

**Table 2:** ^13^C NMR data of pyridoacridine alkaloids **9**–**16**.

	(CDCl_3_/CD_3_OD 2:1)	(DMSO-*d*_6_)				(CDCl_3_/CD_3_OD 2:1)	(DMSO-*d*_6_)	

Position	**9** [47]	**10** [48]	**11** [49]	**12** [50]	**13** [50]	**14** [50]	**15** [50]	**16** [51]

1	131.5	131.0	155.2	132.0	131.3	113.7	115.0	156.1
2	132.1	132.9	115.5	131.1	131.1	135.6	132.0	116.5
3	130.3	131.5	131.8	130.1	130.2	123.1	120.6	133.3
4	123.1	124.5	114.3	124.2	124.4	124.7	124.0	114.5
4a	121.9	122.8	123.5	122.3	121.7	113.7	115.0	123.8
4b	138.1	136.8	136.9	136.3	134.1	128.6	139.4	137.9
5	120.6	121.2	119.8	119.5	154.4	107.4	110.3	121.8
6	149.7	149.4	150.4	148.2	128.4	141.1	151.3	150.2
7a	145.6	142.5	146.9	147.5	146.9	143.2	140.8	147.0
8	180.4	157.7	178.6	178.5	177.9	149.4	149.7	180.0
8a	126.9	99.0	119.4	119.2	119.0	112.8	107.4	130.3
9	149.2	193.5	175.5	158.5	158.5	166.4	168.1	146.0
10	–	34.5	122.4	–	–	–	–	–
11	151.9	40.5	136.7	146.1	144.8	90.1	89.6	147.8
12	116.4	–	–	99.6	99.7	67.7	67.6	123.5
12a	143.5	157.7	146.8	150.2	149.3	110.3	104.4	147.9
12b	145.7	142.6	142.0	147.2	145.5	128.4	128.8	143.1
12c	118.8	116.2	116.9	117.5	118.5	121.6	121.5	120.1
13a	145.1	144.0	133.3	144.7	139.8	141.0	140.2	134.4
MeN-10	–	–	–	37.8	38.0	35.0	35.2	48.3
MeO-5	–	–	–	–	58.0	–	–	–
MeO-11	–	–	–	–	–	–	56.3	–
MeO-12	–	–	–	–	–	–	54.9	–

**Table 3:** ^13^C NMR data of pyridoacridine alkaloids **17**–**19**.

	(150 MHz, CD_3_OD) [51]	(125 MHz, CD_3_CN) [51]	(125 MHz, CDCl_3_/TFA-d) [52]

Position	**17**	**18**	**19**

1	135.8	143.0	126.9
2	124.3	128.4	117.9
3	162.7	133.5	138.1
4	108.0	125.1	133.7
4a	126.6	125.2	114.4
4b	140.9	115.8	122.0
5	122.9	187.2	122.7
6	151.4	71.4	146.0
7a	149.5	115.1	126.2
8	180.6	161.0	174.7
8a	130.8	132.7	113.4
9	147.9	145.9	145.7
11	148.7	142.0	164.2
12	124.1	122.9	115.6
12a	149.2	143.5	142.8
12b	145.5	139.5	142.8
12c	120.8	129.9	111.1
13a	141.5	143.1	144.9
MeN-10	49.3	49.0	39.7
MeN-7	–	54.1	–
MeN-7	–	54.1	–

**Table 4:** ^13^C NMR data of pyridoacridine alkaloids **20**–**26**.

Position	(125 MHz, DMSO-*d*_6_)				(100 MHz, DMSO-*d*_6_)		(100 MHz, CDCl_3_)

	**20** [42]	**21** [40]	**22** [40]	**23** [46]	**24** [53]	**25** [54]	**26** [55]

1	117.5	115.8	116.0	117.3	116.3	132.0	131.5
2	135.3	131.1	131.5	134.4	131.8	131.8	130.1
3	123.0	120.4	120.7	122.5	120.8	131.6	128.3
4	125.5	123.4	123.7	127.1	123.5	124.0	123.0
4a	114.1	115.5	115.7	114.6	115.8	122.5	122.1
4b	148.5	139.0	139.3	152.4	139.4	140.5	138.2
5	107.6	107.9	108.2	107.9	108.4	117.2	114.7
6	142.7	150.4	150.6	144.8	150.9	149.1	149.9
7a	131.8	143.1	143.7	nd	143.5	152.0	149.5
7b	132.7	140.1	140.5	135.3	140.7	140.7	142.0
8	–	–	–	–	–	–	–
9	153.8	148.4	148.6	151.0	148.9	162.1	154.6
10a	143.2	139.1	139.3	144.0	140.0	146.3	148.3
11	108.4	104.3	104.5	107.4	106.0	72.5	74.1
11a	132.8	nd	133.3	133.0	133.8	162.7	161.5
11b	118.3	117.4	117.6	118.2	117.9	115.5	114.0
12a	140.5	139.5	139.7	140.3	139.6	146.5	144.9
13	31.0	30.6	30.9	31.0	34.6	47.2	47.4
14	36.2	36.0	36.2	36.8	39.6	41.5	35.2

	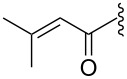	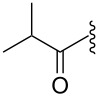	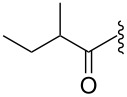	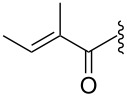	–	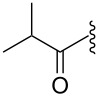	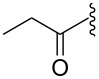

C=O	167.8	176.9	176.8	169.9	–	174.3	173.6
–	118.1 CH	33.6 CH	41.1 CH	131.0 C	–	39.9 CH	29.4 CH_2_
–	150.3 C	19.1 CH_3_	26.5 CH_2_	130.7 CH	–	26.3 CH_3_	9.5 CH_3_
–	26.8 CH_3_	19.1 CH_3_	17.1 CH_3_	13.7 CH_3_	–	26.3 CH_3_	–
–	19.5 CH_3_	–	11.5 CH_3_	12.4 CH_3_	–	–	–

**Table 5:** ^13^C NMR data of pyridoacridine alkaloids **27**–**36**.

Position	(CD_3_OD) [44]	(DMSO-*d*_6_) [42]	(DMSO-*d*_6_) [56]	(DMSO-*d*_6_) [46]	(DMSO-*d*_6_) [47]	(CD_3_OD) [56]		(DMSO-*d*_6_) [49]	(DMSO-*d*_6_) [57]	

	27	28	29	30	31	32	33	34	35	36

1	119.3	117.0	116.9	118.5	117.5	130.0	135.3	131.6	131.1	155.8
2	136.5	133.8	132.7	134.6	125.9	133.9	124.3	132.1	132.9	115.1
3	125.0	122.2	122.2	123.0	133.1	133.1	163.2	130.3	131.6	133.2
4	126.2	124.8	125.0	124.3	127.7	125.3	108.5	124.0	124.7	114.6
4a	116.2	114.7	115.4	115.0	120.7	124.6	127.0	122.5	122.8	123.6
4b	150.8	144.5	142.5	147.0	132.9	138.8	137.8	136.8	136.8	136.9
4c	–	–	–	–	nd	–	–	–	–	–
4d	–	–	–	–	115.5	–	–	–	–	–
5	105.1	105.9	107.3	104.9	112.5	122.2	122.2	118.1	121.3	121.6
6	144.0	146.1	150.5	144.9	148.7	150.8	150.1	147.1	149.5	149.4
7a	124.7	130.6	136.0	129.3	137.3	143.5	143.5	146.7	142.5	142.6
7b	136.6	117.9	121.7	118.2	nd	160.7	160.7	149.6	–	–
8	–	–	–	–	–	–	–	–	157.7	157.6
8a	–	–	–	–	–	–	–	–	99.0	98.9
9	78.9	164.3	164.0	164.1	164.5	41.9	41.8	146.6	193.2	193.4
10	–	–	–	–	–	–	–	121.7	34.6	34.5
10a	139.8	29.5	29.4	30.2	29.9	35.7	35.7	–	–	–
11	108.4	–	–	–	–	194.6	194.9	174.3	40.5	39.9
11a	133.8	128.0	124.4	130.1	114.5	100.4	132.5	122.2	–	–
11b	120.4	–	–	–	113.8	–	–	–	–	–
12	–	111.5	108.4	117.1	112.3	159.0	159.4	178.9	–	–
12a	133.8	131.5	131.5	132.2	–	143.9	139.5	145.6	157.9	15.7
12b	–	117.1	117.1	118.5	–	117.6	117.5	116.9	142.0	139.2
12c	–	–	–	–	–	–	–	–	116.2	116.4
13	29.3	–	–	–	121.3	–	–	–	–	–
13a	–	140.1	140.0	140.8	–	146.2	141.0	145.1	144.1	133.5
14	38.1	28.0	25.8	28.3	–	–	–	–	–	–
14a	–	–	–	–	135.5	–	–	–	–	–
15	–	36.6	36.9	38.0	–	–	–	–	–	–

	–	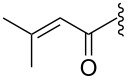	–	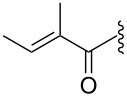	–	–	–	*N*Me 48.9	–	–

C=O	–	168.3	–	171.5	–	–	–	–	–	–
–	–	117.9 CH	–	130.1 C	–	–	–	–	–	–
–	–	150.8 C	–	133.7 CH	–	–	–	–	–	–
–	–	27.0 CH_3_	–	12.2 CH_3_	–	–	–	–	–	–
–	–	19.6 CH_3_	–	14.2 CH_3_	–	–	–	–	–	–

**Table 6:** Structures of selected pyridoacridine alkaloids.

Structures		Ref.

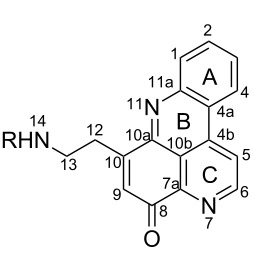	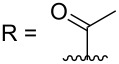 cystodytin J (**1**)	[42]
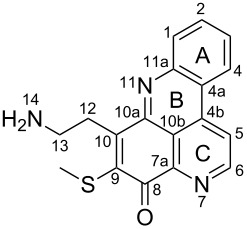	diplamine B (**2**)	[44]
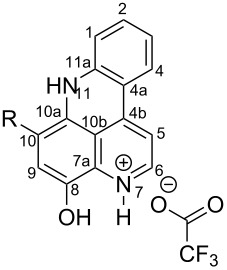	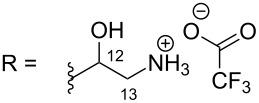 styelsamine A (**3**)	[45]
	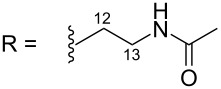 styelsamine B (**4**)	
	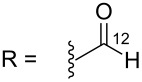 styelsamine C (**5**)	
	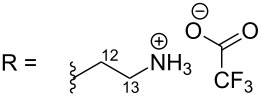 styelsamine D (**6**)	
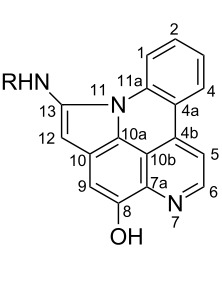	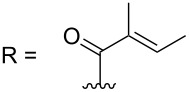 arnoamine C, (**7**)	[46]
	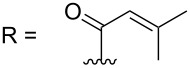 arnoamine D (**8**)	
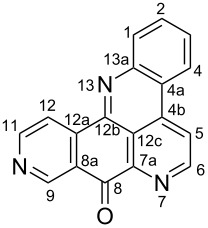	demethyldeoxyamphimedine (**9**)	[47]
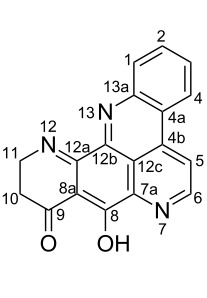	labuanine A (**10**)	[48]
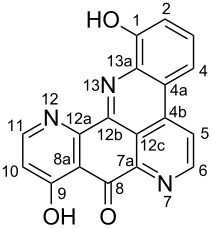	ancorine A (**11**)	[49]
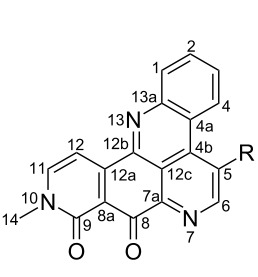	R = H (neoamphimedine, **12**) R = OMe (5-methoxyneoamphimedine, **13**)	[50]
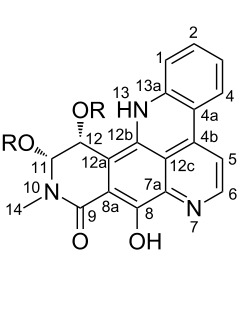	R = H (neoamphimedine Y, **14**) R = Me (neoamphimedine Z, **15**)	
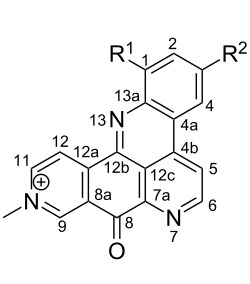	R^1^ = OH, R^2^ = H (1-hydroxydeoxyamphimedine, **16**) R^1^ = H, R^2^ = OH (3-hydroxydeoxyamphimedine, **17**)	[51]
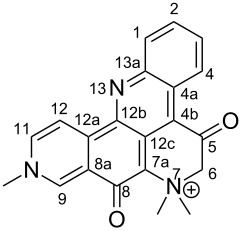	debromopetrosamine (**18**)	
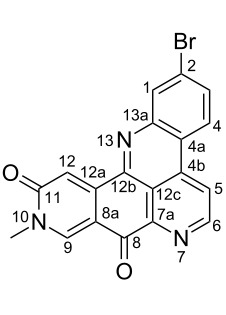	2-bromoamphimedine (**19**)	[52]
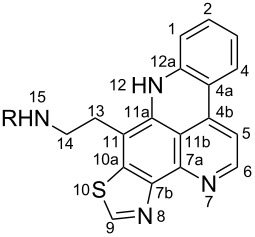	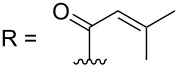 dehydrokuanoniamine B (**20**)	[42]
	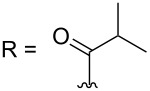 kuanoniamine E (**21**)	[40]
	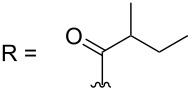 kuanoniamine F (**22**)	[46]
	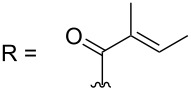 dehydrokuanoniamine F (**23**)	
	R = H (*N*-deacetylkuanoniamine C, **24**)	[53]
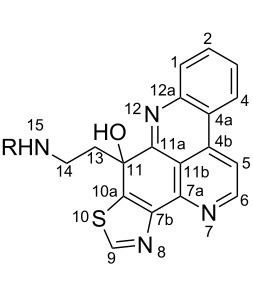	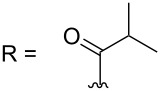 sagitol C (**25**)	[54]
	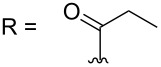 sagitol (**26**)	[55]
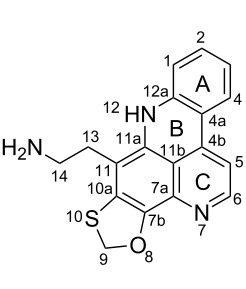	lissoclinidine B (**27**)	[44]
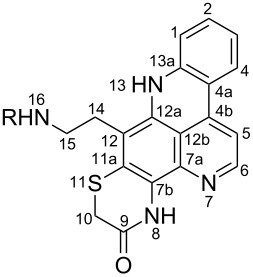	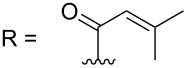 shermilamine C (**28**)	[42]
	R = H (*N*-deacetylshermilamine B, **29**)	[56]
	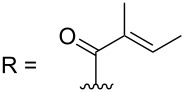 shermilamine F (**30**)	[46]
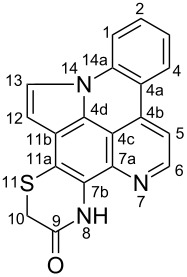	13-didemethylaminocycloshermilamine D (**31**)	[47]
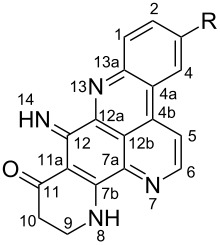	R = H (cystodimine A, **32**)	[56]
	R = OH (cystodimine B, **33**)	
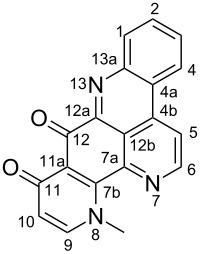	cnemidine A (**34**)	[49]
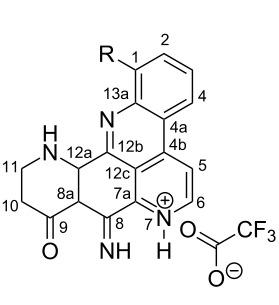	R = H (ecionine A, **35**)	[57]
	R = OH (ecionine B, **36**)	

The chemical shift of the same carbon decreases to 116.7 ppm if the B ring is not aromatic (see compounds **3–8**) [45–46]. Furthermore, a similar feature has been observed with C-6 resonating between δ 148.2 and 151.6 when C-8 bears a ketone (see compounds **2**, **9**, **12**, **13**, and **16**–**19**) [44,50–52] or a β-enol (**10**) [48]. Otherwise, it appears in the range δ 140.6–143.3 if C-8 bears a phenol group (compounds **3**, **4**, **6–8**, **13**, and **14**) [45–46,50].

Likewise, the same downfield resonances have been found for C-6, when C-7b and its neighboring carbons C-10a or C-11a form a thiazole (see compounds **20**–**26**) [40,44,53–55], a thiomorpholinone (compounds **28**–**31**) [42,46–47], a dihydropyridinone (**32** and **33**) [56], or pyridinone (**34**) [49] rings. These rings, bearing a conjugated ketone, presumably extend the electron delocalization to C-6. For compounds **32** and **33**, the downfield shift of C-6 could also be explained by the presence of the imine at C-8 [56]. In addition, the presence of an imine function at C-8 led to the same downfield chemical shift for C-6 (see **35** and **36**) [57]. These observations can be explained either in terms of mesomeric or inductive effects of some functions. The carbon resonances of the *N*-CH_3_ groups in some of these alkaloids could indicate whether or not they are ammonium salts. Thus, carbon atoms of CH_3_ groups attached to ammonium resonate between 48 ppm and 54.1 ppm similar to those of MeO groups (compounds **16**–**18**) [51]. This carbon shift appears around 39.0 ppm (compounds **12**–**15**) if the CH_3_ group is bound to an uncharged nitrogen atom [50].

Rings A–C (Table 6) rarely contain functional groups and the hydrogen chemical shifts (compounds **12**–**14**, **17**, and **19**) can be used as a starting point for structure elucidation. 2D Long range correlations maps (HMBC) can easily lead to a substructure that could be considered complete with the aforementioned observation. Furthermore, the assignment of the ^13^C shifts of a new, isolated metabolite could be made by comparing with those compiled in Table 6, where the mass spectrometry data provides information on the elemental composition leading to the deduction of the structure.

#### Mass fragmentation

**Figure 1:**
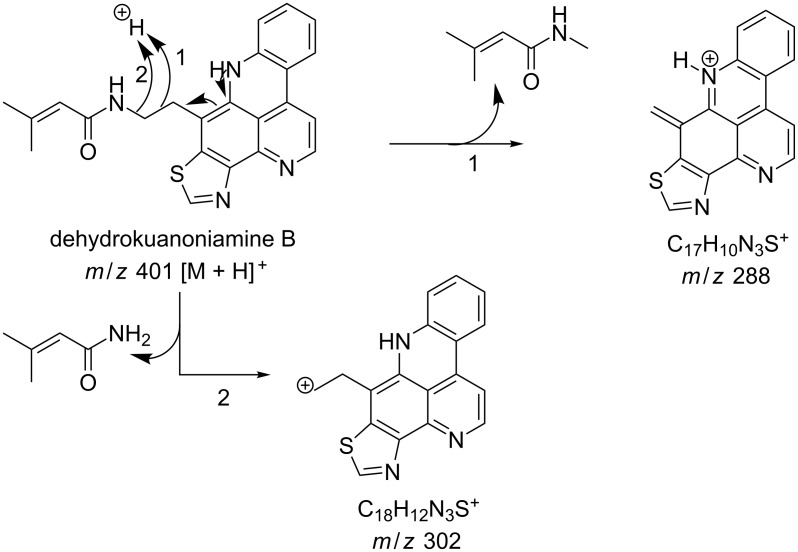
Fragments produced by the FAB–MS of dehydrokuanoniamine B (**20**) [42].

**Figure 2:**
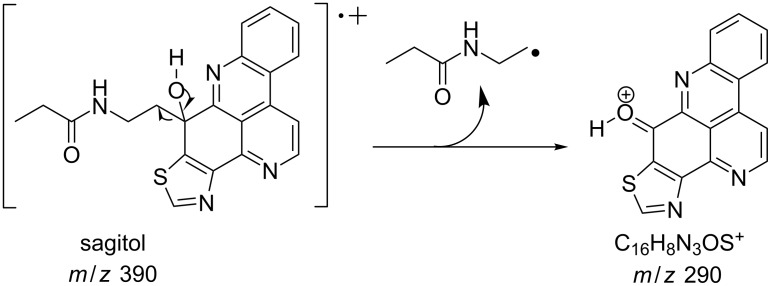
Fragments produced by the EIMS of sagitol (**26**) [55].

**Figure 3:**
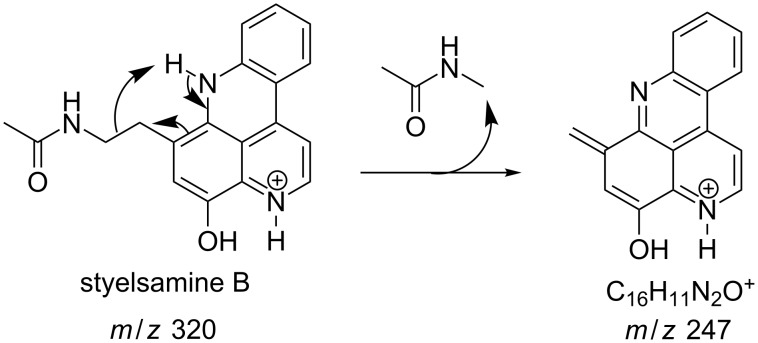
Fragments produced by the EIMS of styelsamine B (**4**) [45].

**Figure 4:**
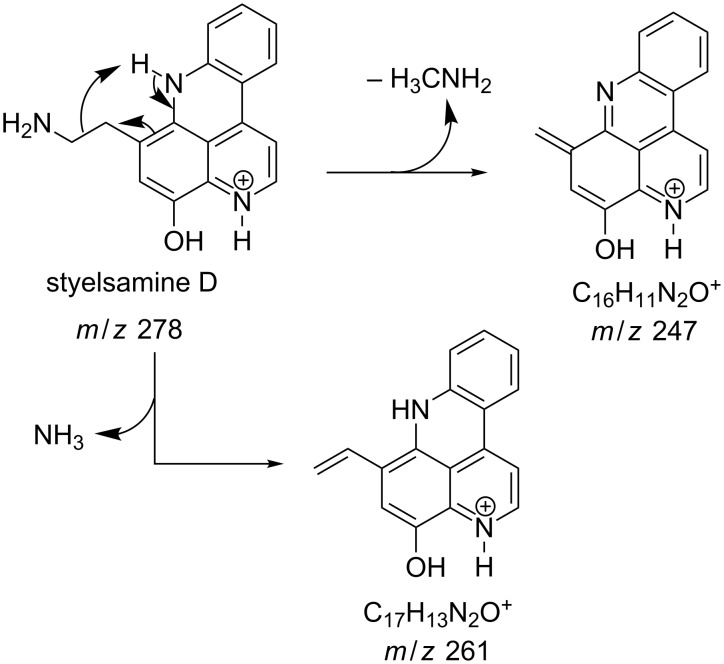
Fragments produced by the EIMS of styelsamine D (**6**) [45].

**Figure 5:**
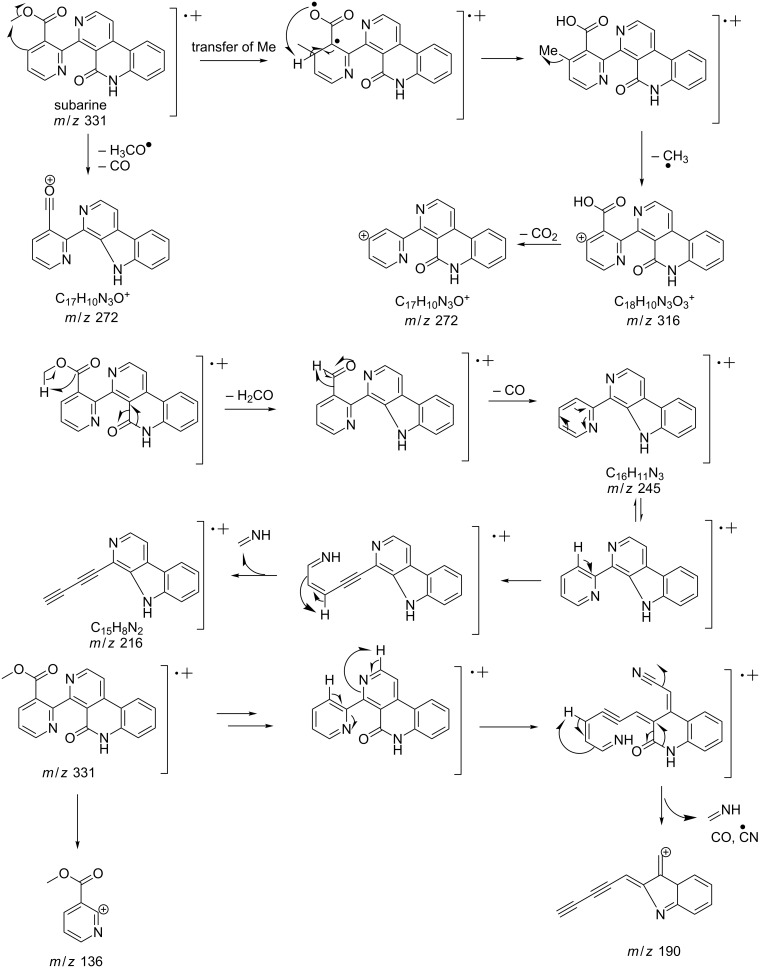
Fragments produced by the EIMS of subarine (**37**) [40].

The mass spectrometry spectrum of pyridoacridines contains very little information due to the lack of fragments. Independent of the ionization source used to determine the elemental composition (ESI, DIC, APCI, FAB, or EI), molecules with compact, and fused rings do not undergo fragmentation and only their corresponding ion peak is observed [47]. However, those containing a side chain show a few ion peaks corresponding to the sequential fragmentation of the latter. This observation is supported by the mass spectrometry data of styelsamine B (**6**) and sagitol (**26**), whose fragments could be used to determine the pyridoacridine skeleton (Figures 1–4) [42,45,55]. The electron-impact mass spectrometry of subarine (**37**) showed good fragmentation because of the partial flexibility of the structure (Figure 5) [40].

### Recent synthesis of pyridoacrydines

Many synthetic routes have been used for the preparation of pyridoacridine alkaloids according to their structural core. However, the number of synthetic pathway steps for their preparation has varied from few to many. In some cases, the preparation of alkaloids such as cystodytin J (**1**), styelsamine B (**4**), demethyldeoxyamphimedine (**9**), and subarine (**37**) was strategically achieved with up to five steps.

#### Synthesis of styelsamine B (**4**) and cystodytin J (**1**)

**Scheme 1:**
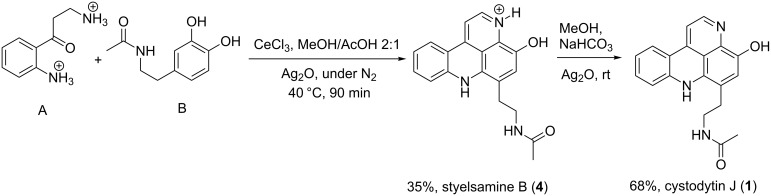
Synthesis of styelsamine B (**4**) and cystodytin J (**1**) [58].

The first route showed the total synthesis of styelsamine B via oxidation, Michael addition, condensation and cyclization reaction steps. Precursors A and B were adequately prepared and used to synthesize **1** and **4** in a one-pot manner in the presence of cesium chloride, silver oxide in methanol and acetic acid under nitrogen atmosphere. The reaction lasted 90 min at 40 °C. The proposed mechanism postulated that silver oxide oxidized the *N*-acetyldopamine (B) into an *O*-quinone derivative which reacted itself with the arylamine (A) through a Michael addition mechanism and the acridine core was formed via a nucleophilic condensation process. The addition step was also facilitated by CeCl_3_ used here as a Lewis acid. The last step, characterized by the imine formation and aromatization, occurred presumably due to the presence of an acidic solvent. Styelsamine B (**4**) was obtained with an overall yield of 35%. This pyridinium salt was treated with an ampholyte to afford its neutral form cystodytin J (Scheme 1) [58].

This biomimetic synthetic strategy is quick and economically accessible for developing styelsamine B (**4**) and cystodytin J (**1**) bioactive derivatives.

#### Synthesis of sebastianine A (**38**) and its regioisomer (**39**)

**Scheme 2:**
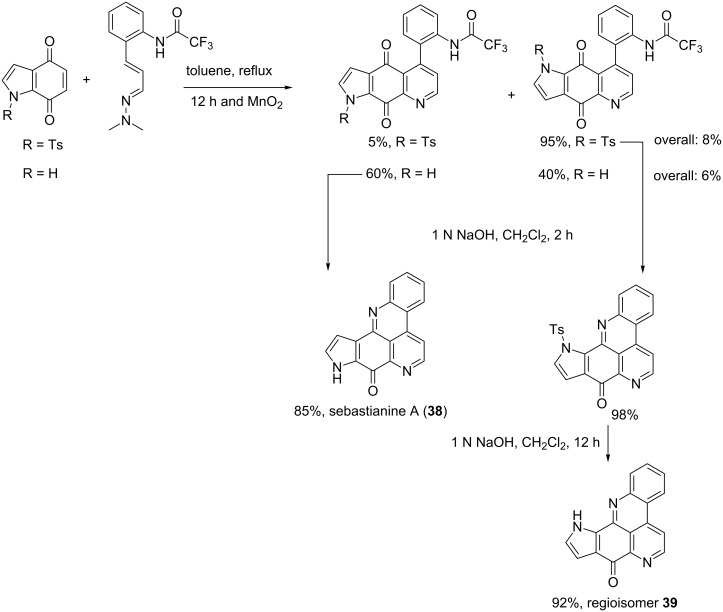
Synthesis of sebastianine A (**38**) and its regioisomer **39** [59].

The first step of the sebastianine A synthesis was based on a Diels–Alder reaction between a pyrroloquinone used as the dienophile and *o*-trifluoroacetamidocinnamaldehyde dimethylhydrazone playing the role of the diene. This medium was refluxed in toluene for 12 h and the product was oxidized with manganese oxide to give two intermediates. The latter were separately subjected to cyclization and deprotection reactions in alkaline conditions to give the natural product and its regioisomer (Scheme 2) [59].

The Diels–Alder cyclisation seems to be the key step for sebastianine A synthesis with a very low yield. Recently, the same cycloaddition was successfully performed on related compounds by attaching a bromine atom on the pyrroloquinone [60]. The halogen presumably increased the electron delocalization in the diene allowing the overlap of molecular orbitals.

#### Synthesis of neoamphimedine (**12**)

**Method A**: To prepare neoamphimedine, 4-methoxy-2,6-dinitrophenol was methylated with diazomethane and the product was partially reduced to the amine by using palladium on carbon (Pd/C) as a catalyst. The *N*-acetyl group was further introduced by treating the product from the reduction with acetic acid and acetic anhydride. Furthermore, a second reduction with Pd/C was performed followed by the reaction of the obtained amine with ethyl (2-nitrobenzoyl)acetate to give a β-keto amide. The resulting amide was subjected to a Knorr cyclization in the presence of polyphosphoric acid to afford a quinolinone, which was further reduced to a quinoline in two steps. The Sandmeyer reaction permitted the replacement of the amide function with a carboxylic function by first hydrolyzing the amide function to the corresponding amine following by the preparation of the diazonium and substitution of the diazonium function with a nitrile group. The latter was then converted to a carboxylic group under strongly acidic conditions.

**Scheme 3:**
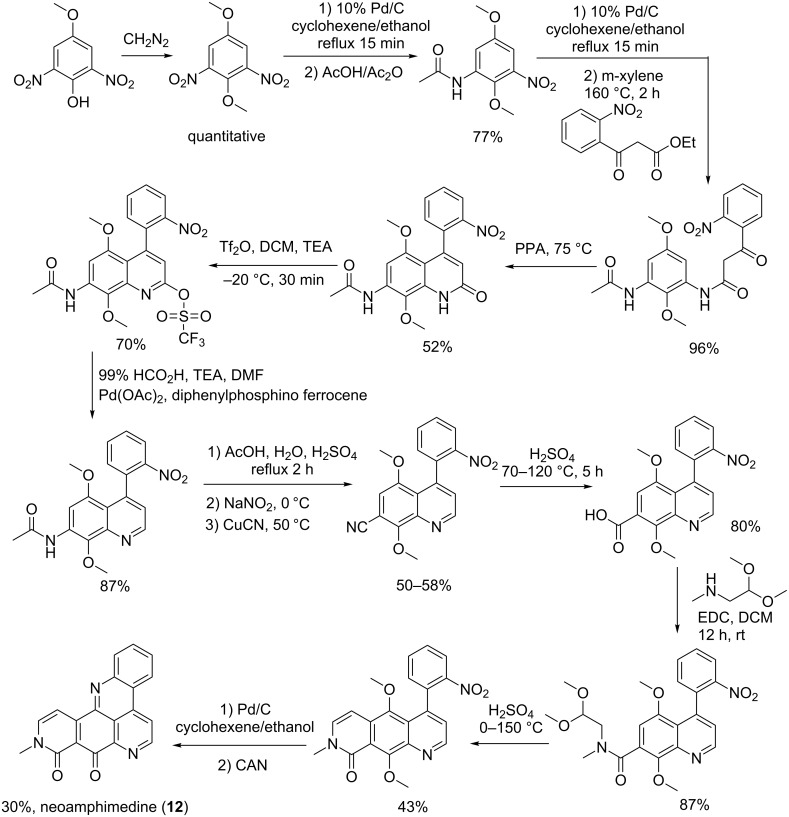
Synthesis route A of neoamphimedine (**12**) [61].

The acid derivative was treated with methylaminoacetaldehyde dimethylacetal and cyclized to form an isoquinoline ring. The nitro group in that intermediate was reduced following a one-pot oxidation and cyclization to afford the target molecule (Scheme 3) [61].

**Scheme 4:**
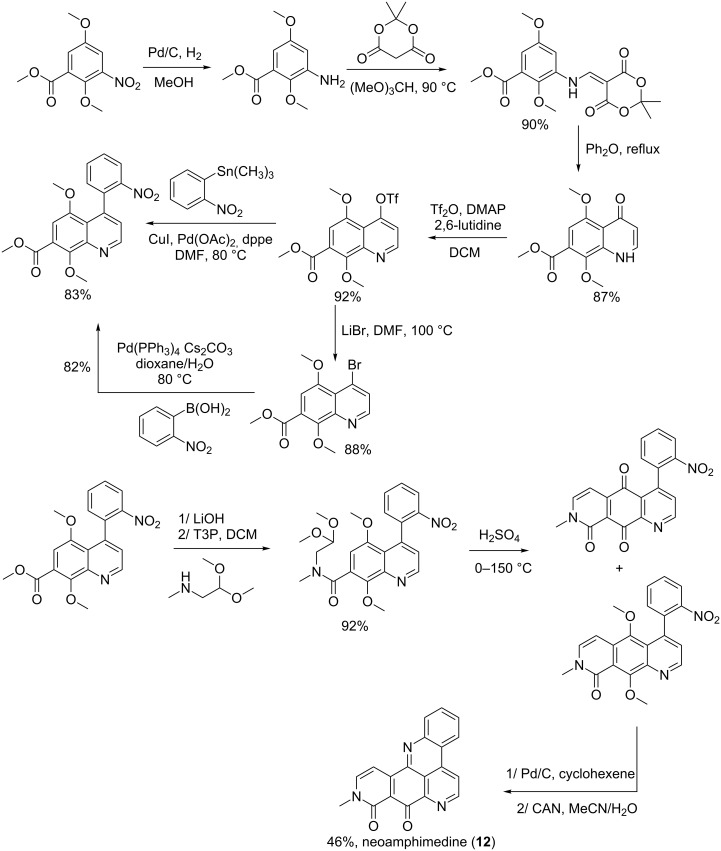
Synthesis route B of neoamphimedine (**12**) [62].

**Method B:** Neoamphimedine was prepared by Li et al. [62] in eight and nine steps with a 25% overall yield. The reaction started with methyl 2,5-dimethoxy-3-nitrobenzoate, which was reduced into the corresponding aniline under hydrogen conditions with palladium on carbon. The aniline was further treated with Meldrum’s acid and trimethyl orthoformate to afford an enamine which was subsequently transformed into a quinolone benzyl ether under reflux conditions. A quinoline triflate ester was prepared from the quinolone and coupled to trimethyl(2-nitrophenyl)stannane by Stille coupling catalyzed by palladium acetate. The afforded nitrophenylquinoline was hydrolyzed and converted into an amide in the presence of methylaminoacetaldehyde dimethylacetal. A ring closure occurs by treating the amide with a strong acid. The reaction gave a mixture of a quinone and a dimethoxy intermediate that were first reduced to amine products. Furthermore, the dimethoxy intermediate was subjected to cerium ammonium nitrate for oxidative demethylation and the amino quinone formed cyclized to afford neoamphimedine (**12**, Scheme 4) [62].

Two synthetic routes, illustrated in Scheme 2 and Scheme 3, were used to prepare neoamphimedine (**12**) where the second description was shorter and more efficient than the first. Other differences have been noted such as Friedel–Craft acylation in sulfuric acid that led to one product after chromatographic column purification, while in the second preparation, a mixture of two adducts was obtained after an absorbent-free purification. This may explain why the yield of **12** was greater in the second preparation. Nevertheless, the red color mentioned for neoamphimedine after route B seems unusual. The natural product is known as a yellow solid [61] suggesting that the neamphimedine in this case might be contaminated with ceric ammonium nitrate (CAN), which has an orange-red color.

#### Synthesis of arnoamines A (**40**) and B (**41**)

**Scheme 5:**
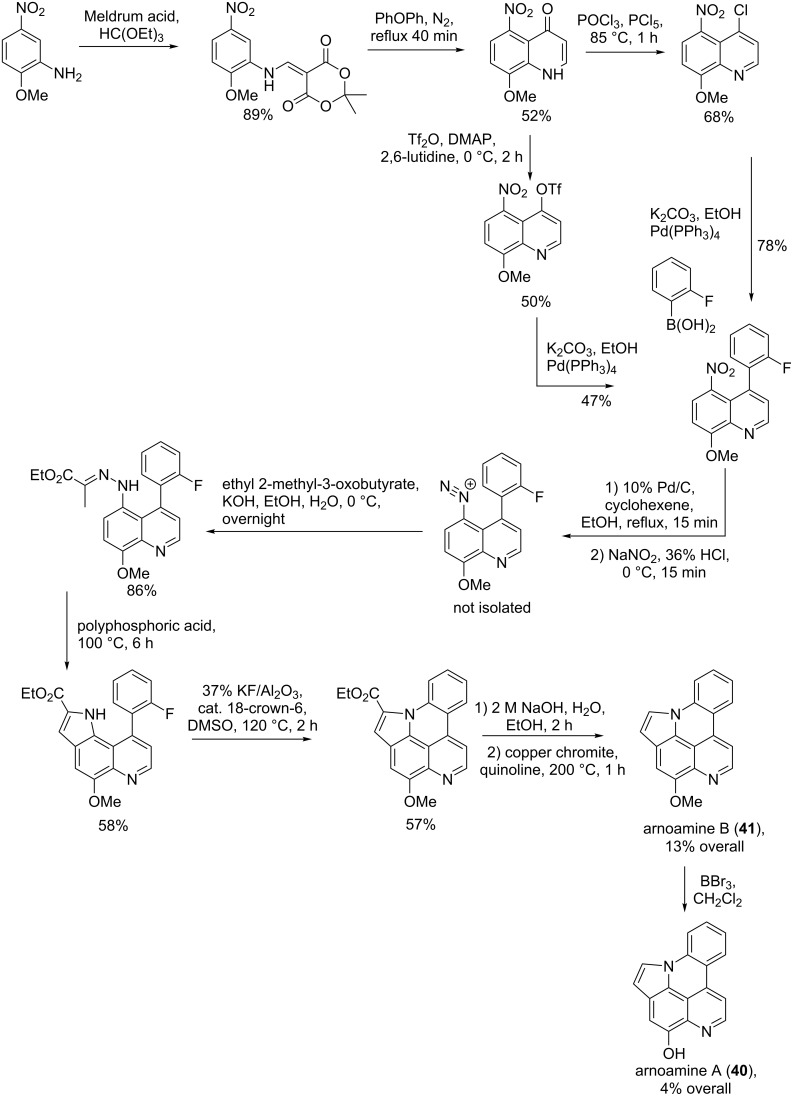
Synthesis of arnoamines A (**40**) and B (**41**) [63].

The synthesis of arnoamines A and B was accomplished in nine and ten steps in 13% and 4% overall yield, respectively. The reaction started with 2-methoxy-5-nitroaniline treated with Meldrum’s acid (2,2-dimethyl-1,3-dioxane-4,6-dione) and ethyl orthoformiate to afford an enamine derivative, which was further cyclized in the presence of biphenyl ether under nitrogen atmosphere and reflux conditions for 40 min. A quinolinone was obtained in 52% yield and subjected to two different reactions, firstly with phosphoryl chloride and phosporus pentachloride to prepare 4-chloro-8-methoxy-5-nitroquinoline and secondly with triflate anhydride, dimethylaminopyridine, and 2,6-lutidine to prepare 8-methoxy-5-nitro-4-triflylquinoline. The Suzuki cross-coupling was carried out on both quinoline derivatives in the presence of a palladium catalyst and the appropriate organoborane to give the expected product with 47 and 78% yield for the chloride and triflate substrates, respectively. The nitro function present in the product was then reduced by palladium-catalyzed hydrogenation to yield the amine product converted into a diazonium salt. A Japp–Klingemann reaction was used to transform the diazonium salt in the presence of ethyl 2-methyl-3-oxobutyrate into the respective hydrazone. The indole was formed from the treatment of the hydrazone with polyphosphoric acid. The last heterocyclic ring was formed by an intramolecular reaction of the indole catalyzed by potassium fluoride in alumina and a sufficient amount of 18-crown-6. The product obtained from this reaction was decarboxylated by copper chromite and quinolone to yield arnoamine B. This latter was then demethylated with boron tribromide to afford arnoamine A (**40**, Scheme 5) [63].

#### Synthesis of ascididemin (**42**)

**Scheme 6:**
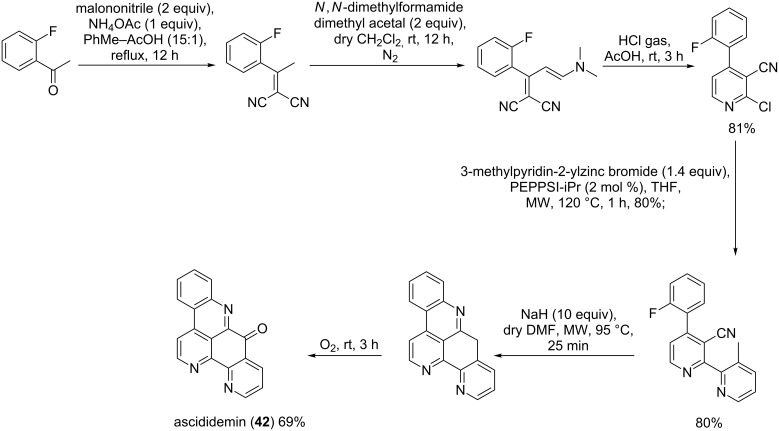
Synthesis of ascididemin (**42**) [65].

The first synthesis of ascididemin was performed in 1992 by Moody et al. [64] and included four steps with an overall yield of 21%. Recently, the same secondary metabolite was prepared by six efficient steps which afforded a yield two-fold (45%) of that of the previous synthesis [65]. This preparation started with a Knoevenagel condensation of 2-fluoroacetophenone with malononitrile. The product of the condensation reacted with an excess of *N*,*N*-dimethylformamide dimethyl acetal to afford an enamine. This latter was treated with hydrochloric acid in acetic acid gave 4-aryl-2-chloro-3-cyanopyridine, which was cross-coupled with 3-methylpyridin-2-ylzinc bromide catalyzed by PEPPSI-iPr (pyridine-enhanced precatalyst preparation stabilization and initiation or 1,3-bis(2,6-diisopropylphenyl)imidazol-2-ylidene](3-chloropyridyl)palladium(II) dichloride) under microwave conditions. The bipyridine derivative obtained was subjected to a strong base (sodium hydride) to give 12-deoxyascididemin which was immediately oxidized in situ by oxygen to yield ascididemin (**42**, Scheme 6) [65].

#### Synthesis of subarine (**37**)

**Scheme 7:**
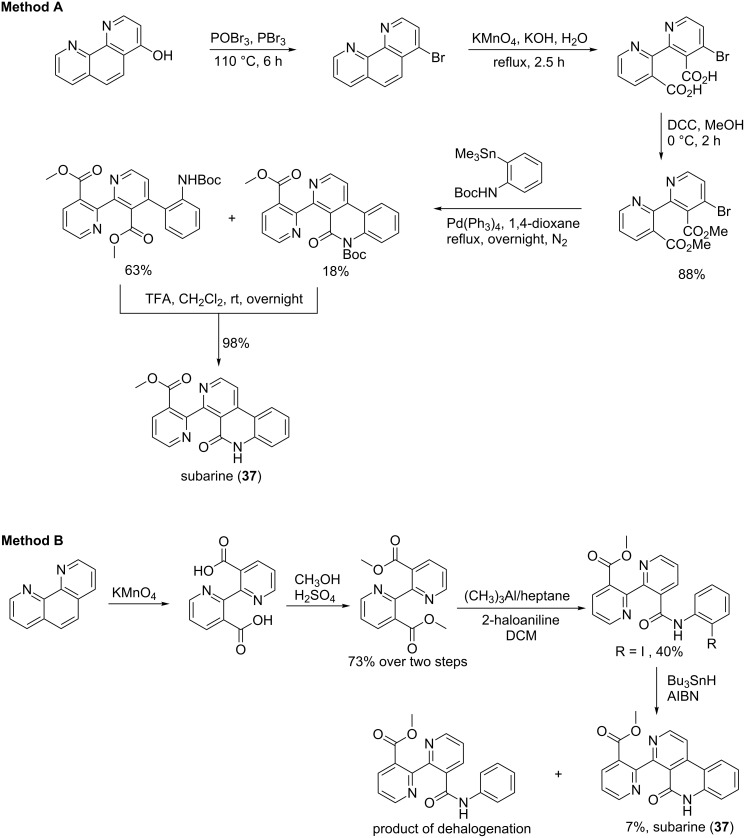
Synthesis of subarine (**37**) [66–67].

**Method A:** This alkaloid was successfully prepared in five steps with 70% overall yield. The synthesis started with 4-bromo-1,10-phenanthroline subjected to an oxidative cleavage with potassium permanganate to afford binicotinic acid. The binicotinic acid product was esterified using dicyclohexylcarbodiimide (DCC) in methanol and the resulting ester was cross-coupled to *N-(tert*-butoxycarbonyl)-2-(trimethylstannyl)aniline using Stille conditions. The expected compound was obtained along with the *N*-Boc-protected subarine. The treatment of both compounds with trifluroacetic acid gave subarine (**37**, Scheme 7) [66].

**Method B:** A second synthesis path of subarine (**37**) has been performed by Lotter and Bracher [67]. The route included four steps, but unfortunately, the overall yield was only 7%. Like in method A, the synthesis started with 1,10-phenanthroline to prepare binicotinic acid via oxidative cleavage by potassium permanganate. The esterification took place by treating the binicotinic acid with methanol and sulfuric acid. The resulting product was transformed into a 2-haloanilide under Weinreb conditions, namely trimethylaluminium and 2-haloaniline. Subarine (**37**) was subsequently obtained from the radical cyclization of the haloanilide derivative in the presence of tributyltin hydride and azobisisobutyronitrile (AIBN) (Scheme 7) [67].

Two methods were reported for the synthesis of subarine (**37**) where the first (method A) is a versatile path based on the outcome yield. The key step in method A is the Stille cross-coupling characterized by a transmetalation, while in method B, the cyclisation was performed in a radical mechanism manner. Whereas the radical condition afforded a low yield of the expected product, the Stille coupling seems to be a better solution.

#### Synthesis of demethyldeoxyamphimedine (**9**)

**Scheme 8:**
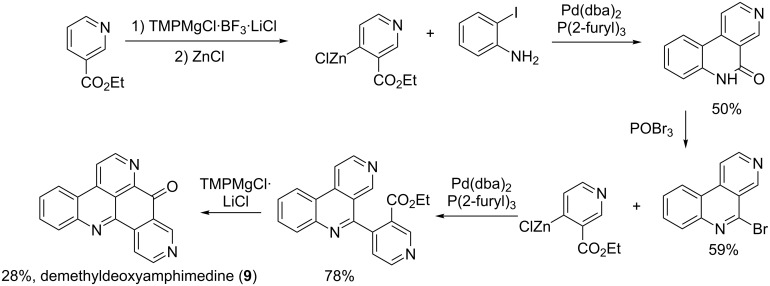
Synthesis of demethyldeoxyamphimedine (**9**) [68].

The synthesis of demethyldeoxyamphimedine was successfully performed in six steps with an overall yield of 6.5%. The steps included an organometallic intermediate and a Negishi cross-coupling reaction characterized by a transmetalation with zinc and palladium. The benzonaphthyridinone product was transformed into a bromobenzonaphthyridine intermediate by using phosphoryl bromide. Another organozinc substrate was coupled to the obtained intermediate by a Negishi cross-coupling and cyclisation occurred to give the expected secondary metabolite (Scheme 8) [68].

The yield of the last step in the preparation of **9** could be improved by using the synthetic protocol previously reported for a similar ketone prepared from pyridine and *N*,*N*-dimethylbenzamide [69]. The first sequence of the route was the metalation of pyridine subsequently using BuLi-LiDMAE, then treating pyridyllithium formed with *N*,*N*-dimethylbenzamide. The ketone was recovered with 80% within 2 h. Therefore, the second Negishi coupling needed to be performed with *N*,*N*-dimethylnicotinamidezinc chloride.

#### Synthesis of pyridoacridine analogues

**Scheme 9:**
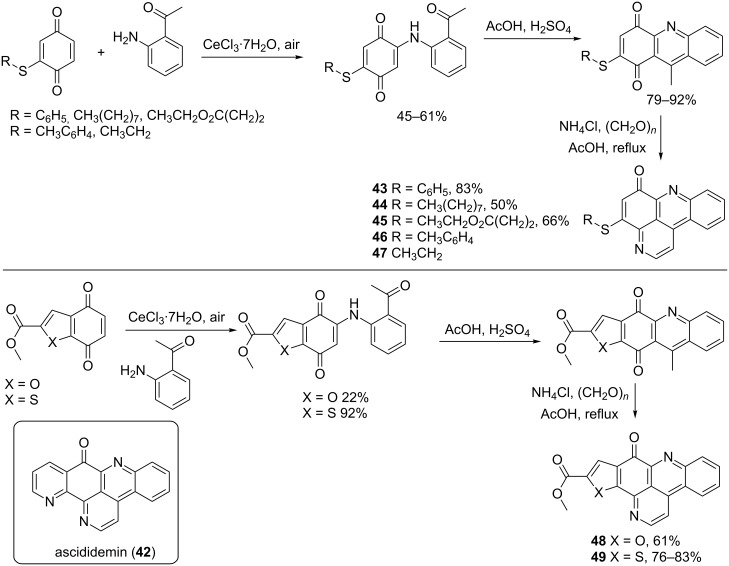
Synthesis of pyridoacridine analogues related to ascididemin (**42**) [70].

The strong antiproliferative activities shown by these alkaloids inspired the design and the synthesis of many pyridoacridines analogues. Scheme 9 shows the synthesis of derivatives **43**–**49**, which imitate the ascididemin (**42**) structure. Compounds **43**–**45** have one pyridine ring less and do not contain the quinolinophenanthroline core as ascididemin. The pyridine ring missing in the structures of **43**–**45** has been replaced in **48** and **49**, by a thiophene and a furan ring, respectively (Scheme 9) [70].

**Scheme 10:**
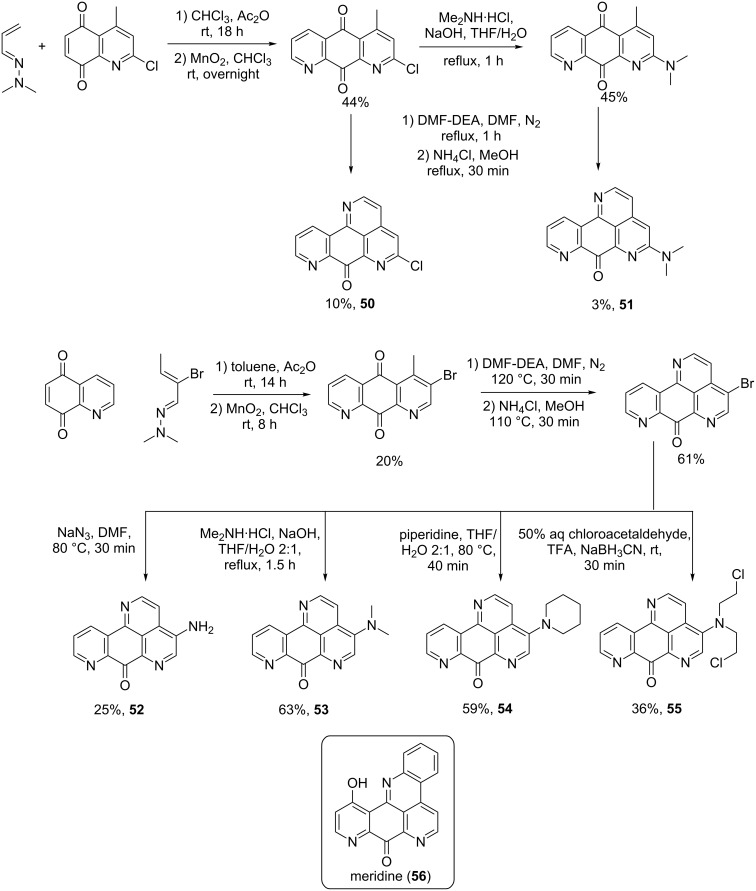
Synthesis of analogues of meridine (**56**) [71].

Analogues (**50**–**55**) shown in Scheme 10 are based on the meridine (**56**) structure but contain a pyridophenanthrolinone scaffold instead of a benzopyridophenanthrolinone as the natural product. In addition they have a tetracyclic core instead of being pentacyclic such as meridine (**56**) (Scheme 10) [71].

**Scheme 11:**
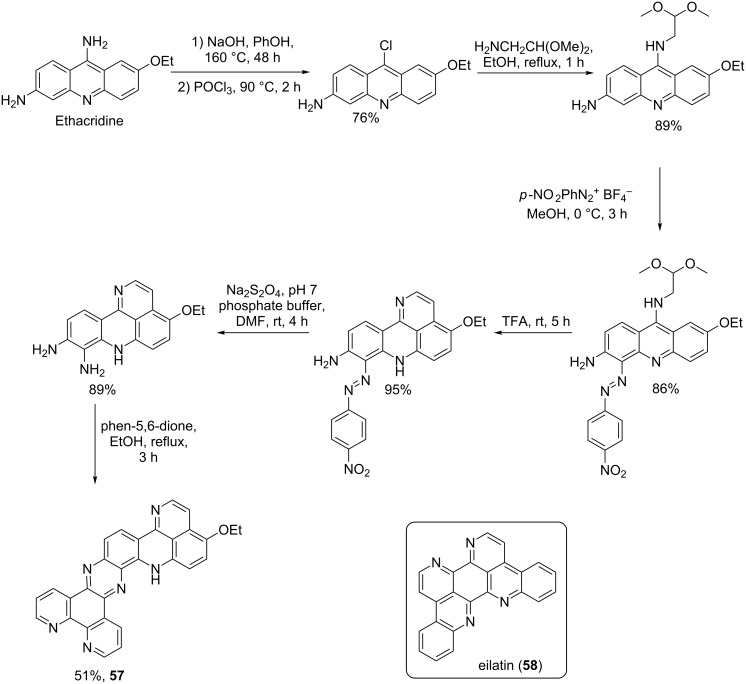
Synthesis of bulky pyridoacridine as eilatin (**58**) [72].

An octacyclic alkaloid (**57**) was also prepared and its synthesis is illustrated in Scheme 11 [72]. Eilatin (**58**) inspired the synthesis of **57** but the compounds do not have structural similarities apart from their bulky size.

**Scheme 12:**
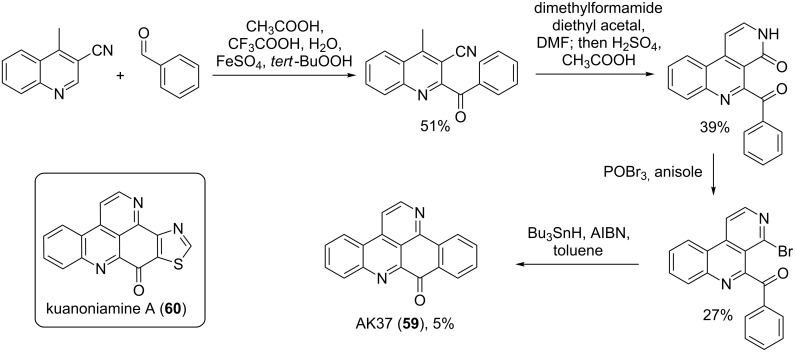
Synthesis of AK37 (**59**), analogue of kuanoniamine A (**60**) [73].

Kuanoniamine A analogue **59** was synthesized as shown in Scheme 12. Its structure differs from that of the natural product **60** by the substitution of the thiazole ring in kuanoniamine A (**60**) with an aryl ring (Scheme 12) [73].

#### Biosynthesis

**Figure 6:**
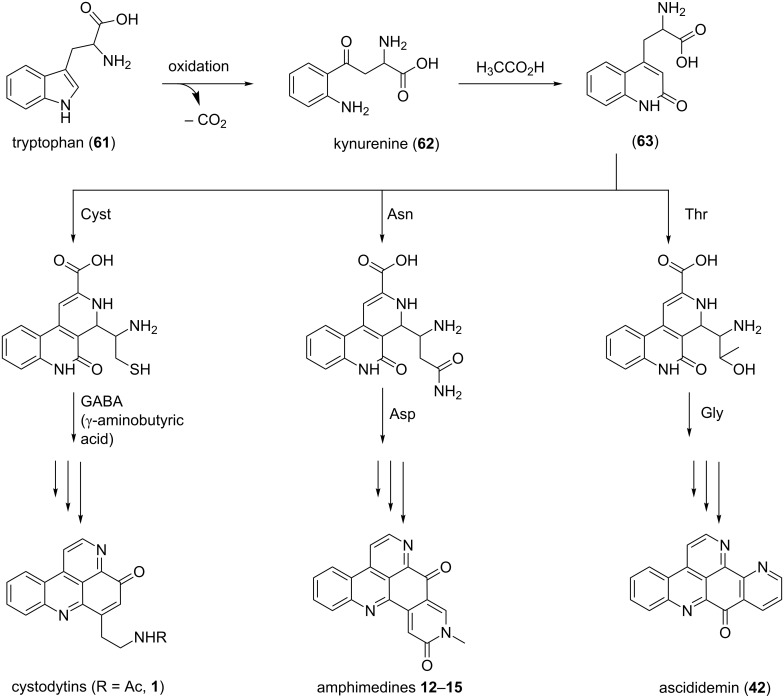
Biosynthesis pathway I [74].

The biosynthesis of pyridoacridine alkaloids has been poorly investigated and reported with only a few studies performed on this topic. Riddick showed the oxidation product of tryptophan (**61**) (kynurenine (**62**)) as the precursor for the biosynthesis of pyridoacridines (Figure 6) [74]. Kynurenine (**62**) with acetic acid forms the quinolinone **63**, which in presence of amino acids such as cysteine, asparagine, threonine, glycine or γ-aminobutyric acid gave different types of these marine alkaloids.

**Figure 7:**
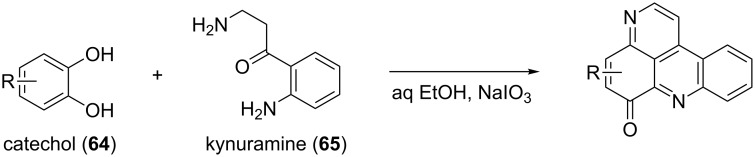
Reaction illustrating catechol and kynuramine as possible biosynthetic precursors [75].

Previously, Gellerman demonstrated (Figure 7) that catechol (**64**) and kynuramine (**65**) could be potential precursors in the eilatin (**58**) (or other pyridoacridines) biosynthesis [75].

**Figure 8:**
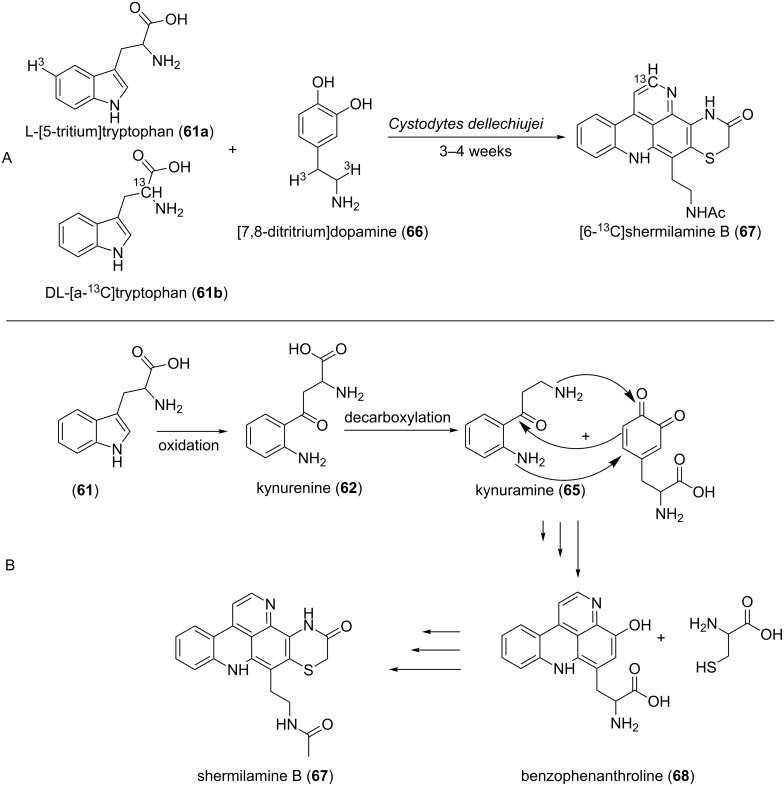
Biosynthesis pathway B deduced from the feeding experiment A using labelled precursors [76].

The feeding experiments with labelled tryptophan (**61a**) and dopamine (**66**) performed by Steffan et al. established these two chemical entities as precursors of shermilamine B (**67**), a secondary metabolite produced by tunicates. Consequently, the authors postulated that tryptophan was transformed into kynurenine (**62**), which was in turn decarboxylated to give kynuramine (**65**). The latter, by reaction with dopamine, forms the benzo-3,6-phenanthroline intermediate **68**, which in turn gave shermilamine B (**67**) upon reaction with cysteine (Figure 8) [76].

**Figure 9:**
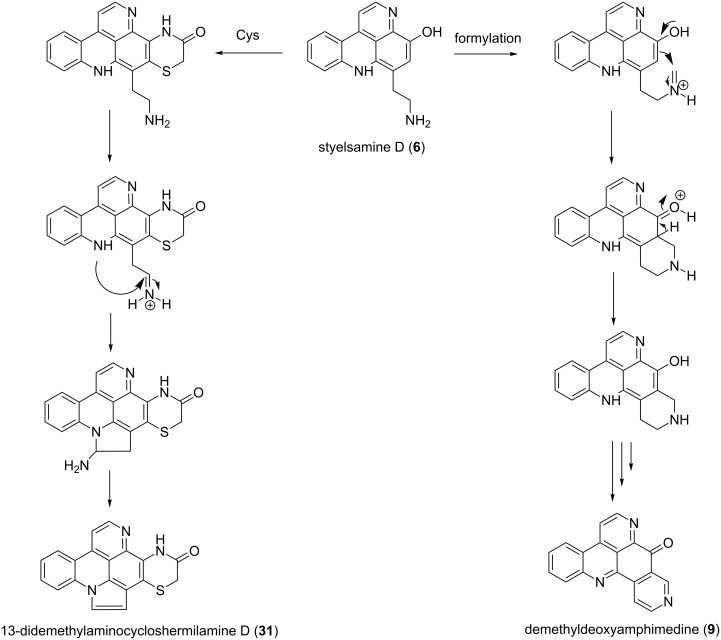
Proposed biosynthesis pathway [47].

The compounds 13-didemethylaminocycloshermilamine D (**31**) and demethyldeoxyamphimedine (**9**) (Figure 9) were presumably formed by reaction of a related benzophenanthroline (styelsamine D, **6**) with cysteine and formaldehyde, respectively [47]. This reaction was followed by cyclization and oxidation to afford the alkaloids (Figure 9).

The analysis of different biosynthesis pathways clearly suggested tryptophan and dopamine as precursors of pyridoacridines. Thus, formaldehyde and amino acids are responsible for the thiazole, piperidone, oxathiolane and thiomorpholinone rings found in pyridoacridines structures.

### Biological activity

The biological activity of pyridoacridines, including both natural and synthetic compounds, has been widely investigated. Many of them displayed cytotoxic activity in addition to other bioactivities such as antiviral, antifungal, antibacterial, antitumor and antiparasitic potential [41]. Several reviews on pyridoacridine alkaloids have been published between 1983–2015 [35,43] that summarize their bioactivity. Herein, a synopsis of the newly published bioactivity of pyridoacridine will be provided as well as pharmacophores associated with the activity and a discussion on the evolution of the bioactivity and the structure modification.

#### Cytotoxicity

**Figure 10:**
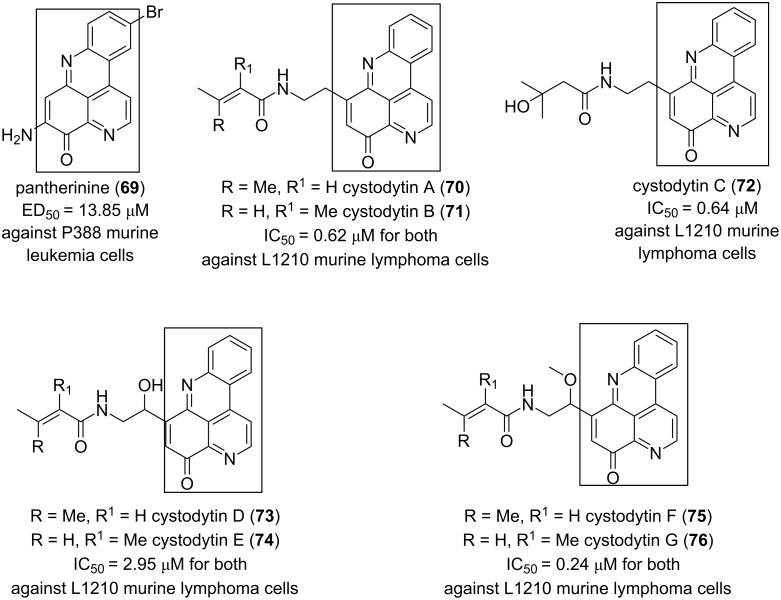
4*H*-Pyrido[2,3,4-*kl*]acridin-4-one as a cytotoxic pharmacophore.

The biologically tested pyridoacridines interestingly displayed strong (IC_50_ < 10 µM) cytotoxic activity in vitro. For instance, pantherinine (**69**) isolated from *Aplidium pantherinum* [77] and cystodytins A–G (**70**–**76**) from *Cystodytes dellechiajei* [36,78] are all potent anticancer metabolites. Their structures are based on a 4*H*-pyrido[2,3,4-*kl*]acridin-4-one scaffold which could be considered as the pharmacophore. The cytotoxicity of **69**–**76** changes depending on the substituents attached to the benzoquinone moiety (Figure 10).

**Figure 11:**
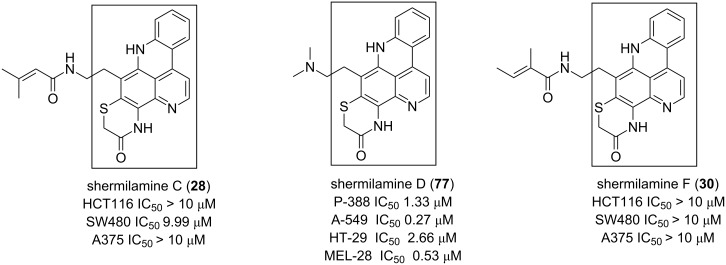
7*H*-Pyrido[2,3,4-*kl*]acridine as a cytotoxic pharmacophore.

Furthermore, shermilamines C (**28**), D (**77**) and F (**30**) also represent one of the interesting anticancer alkaloids. Its structural motif, 7*H*-pyrido[2,3,4-*kl*]acridine fusing with a 2*H*-1,4-thiazin-3(4*H*)-one ring (Figure 11) seems to be less potent than that of **62** [46,79–80].

**Figure 12:**
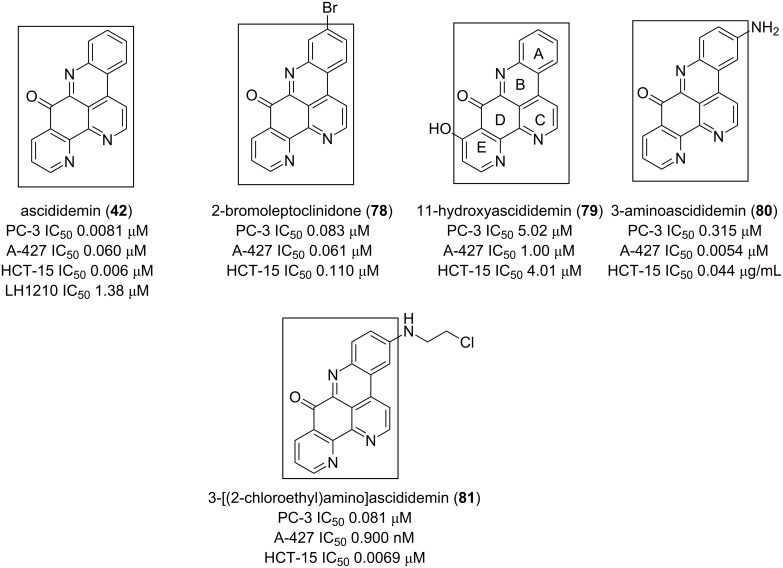
9*H*-Quinolino[4,3,2-*de*][1,10]phenanthrolin-9-one as a cytotoxic pharmacophore.

Ascididemin (**42**) demonstrated interesting antiproliferative activitiy and its pharmacophore 9*H*-quinolino[4,3,2*-de*][1,10]phenanthrolin-9-one remains a versatile core to be explored in cancer chemotherapy [81]. This alkaloid loses its potency when the rings A and E contain a substituent as found in the compounds of **78** and **79**, respectively. The cytotoxic potency is almost recovered when there is a halogenated *N*-alkyl group or an amine function at C-3 (**80** and **81**, Figure 12) [81].

**Figure 13:**
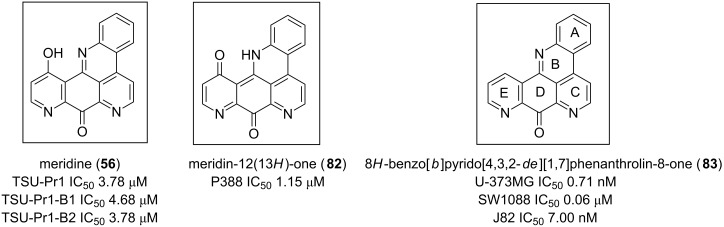
8*H*-Benzo[*b*]pyrido[4,3,2-*de*][1,7]phenanthrolin-8-one as a cytotoxic pharmacophore.

Meridine (**56**) has shown moderate cytotoxicity against an array of cancer lines; nevertheless, it remains a candidate for the design of new and useful anticancer candidates. Some of its reported analogues have shown interesting cytotoxicity against various cancer cell lines [57,69]. As shown in Figure 13, the cytotoxic activity slightly improves when the OH group of the E ring of **56** is oxidized to afford **82** [82]. This bioactivity significantly increases with more selectivity without any substituent on the 8*H*-benzo[*b*]pyrido[4,3,2-*de*][1,7]phenanthrolin-8-one core **83** [83].

**Figure 14:**
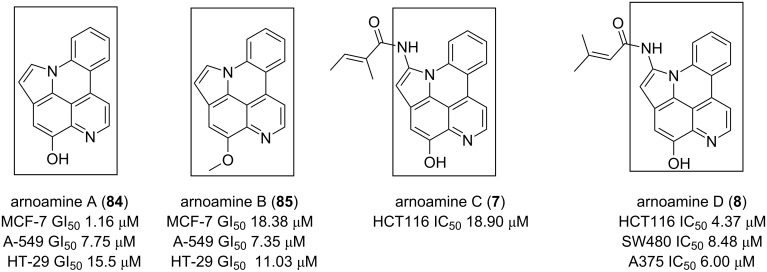
Pyrido[4,3,2-*mn*]pyrrolo[3,2,1-*de*]acridine as a cytotoxic pharmacophore.

Arnoamines A–D (**7**, **8**, **84**, and **85**) isolated from the ascidian *Cystodytes violatinctus* displayed moderate cytotoxicity towards cancer lines HCT116, SW480, and A375 [46]. This alkaloid contains a pyrido[4,3,2-*mn*]pyrrolo[3,2,1-*de*]acridine core closely related to cystodytins. The presence of different substituents (arnoamines A–D, Figure 14) does not significantly change the activity and they seem less active than their congeners, cystodytins (**70**–**76**) [84].

**Figure 15:**
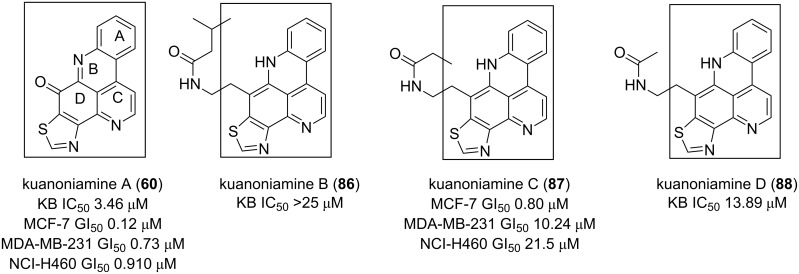
9*H*-Pyrido[4,3,2-*mn*]thiazolo[4,5-*b*]acridin-9-one and 8*H*-pyrido[4,3,2-*mn*]thiazolo[4,5-*b*]acridine: cytotoxic pharmacophores.

Kuanoniamine A (**60**), an alkaloid obtained from the marine sponge *Oceanapia sagittaria,* was found to be a potent growth inhibitor of tumor cells, causing an extensive reduction of the MCF-7 breast cancer cells in the G2/M phase [85]. Two skeletons, including 9*H*-pyrido[4,3,2-*mn*]thiazolo[4,5-*b*]acridin-9-one and 8*H*-pyrido[4,3,2-*mn*]thiazolo[4,5-*b*]acridine (Figure 15), are found in kuanoniamine structures (**60**, **86**–**88**). The first one associated with **60** is more cytotoxic than the reduced alkylated form found in kuanoniamines (B–D) (**86**–**88**) [85–86].

Although all the pyridoacridines have not been tested on the same cancer cell lines, 9*H*-quinolino[4,3,2*-de*][1,10]phenanthrolin-9-one represents the most cytotoxic pharmacophore with a large selectivity according to the reported IC_50_ data. A linear or angular arrangement, as found in ascididemin and neoamphimedine structures, respectively, are two interesting backbones that could be used as starting points in the search for new anticancer drugs. Pyridoacridones containing fused rings with 1,4-quinone somehow showed cytotoxic activitiy although the lack of a pyridine E ring or the presence of a thiazole and a thiazinone ring diminished the activity. A similar loss of potency is also observed when the pyridine E ring contains substituents like MeO and OH groups.

#### Antimicrobial activity

An interesting antimicrobial potency (minimal inhibitory concentration, MIC, below 10 µg/mL) has been assigned to some pyridoacridines such as meridine (**56**) and ascididemin (**42**). Compound **56** inhibited the growth of *Candida albicans* (MIC: 0.2–3.1 µg/mL) and *Cryptococcus neoformans* (MIC 0.8 µg/mL) as well as that of *Trichophyton mentagrophytes* (MIC 6.2 µg/mL) and *Epidermophyton floccosum* (MIC 1.6 µg/mL) [87]. Though no activity was observed against the gram negative bacteria *Escherichia coli* and *Pseudomonas aeruginosa,* meridine (**56**) significantly inhibited the growth of the gram positive bacteria *Bacillus subtilis,* giving a MIC of 3.1 µg/mL [87]. **42** displayed in vitro antiparasitic activity against *Plasmodium falciparum* (K1, NF54), *Leshmania donovani*, *Trypanosoma cruzi* and *T. rhodesiense* but the effect was much lower than that of standard drugs artemisinin and chloroquine [88].

**Figure 16:**
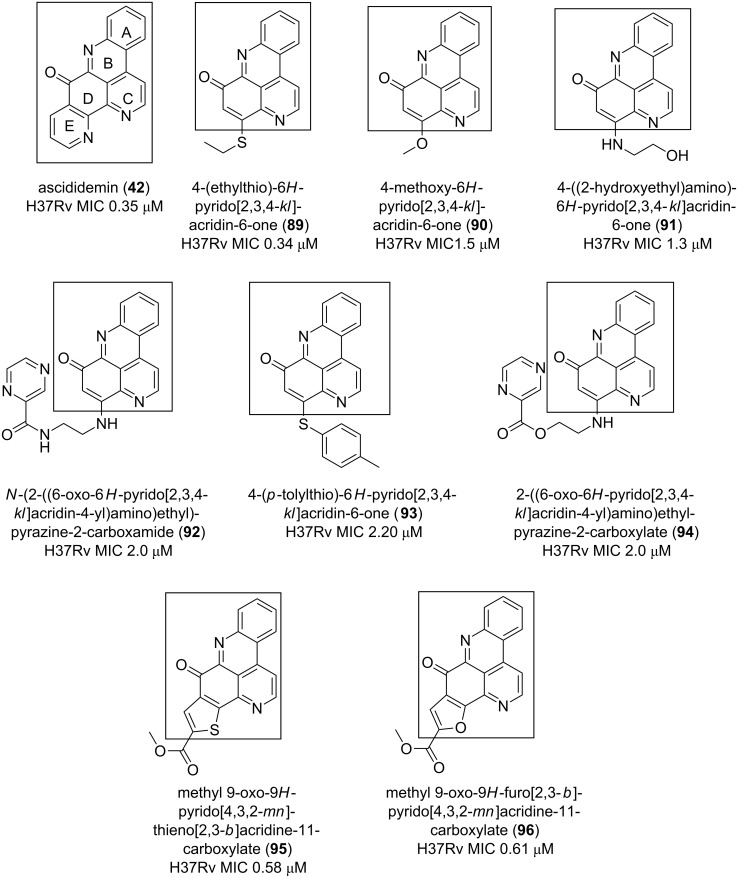
9*H*-quinolino[4,3,2-*de*][1,10]phenanthrolin-9-one as an anti-mycobacterial pharmacophore.

Ascididemin (**42**) displayed significant antituberculosis (TB) activity (MIC of 0.35 µM) against *Mycobacterium tuberculosis* H37*Rv* [70]. The 6*H*-pyrido[2,3,4-*kl*]acridin-6-one motif has been used as a core to develop other anti-TB compounds. In this way, the same activity has been observed with 4-(ethylthio)-6*H*-pyrido[2,3,4-*kl*]acridin-6-one (**89**) against the strain H37Rv with a MIC of 0.34 μM. The anti-TB activity decreases by a factor of 4 to 7 when the thioethyl group at position 8 is replaced by other functionalities (compounds **90**–**94**) (Figure 16).

Other synthetic analogues with skeletons (9*H*-pyrido[4,3,2-*mn*]thieno[2,3-*b*]acridin-9-one **95**; 9*H*-furo[2,3-*b*]pyrido[4,3,2-*mn*]acridin-9-one, **96**) related to that of ascididemin (**42**) [70] also displayed antibacterial activity two-fold lower than that of **42** and **89**. Nevertheless, both **95** and **96** remain good candidates such as **89** to be explored.

**Figure 17:**
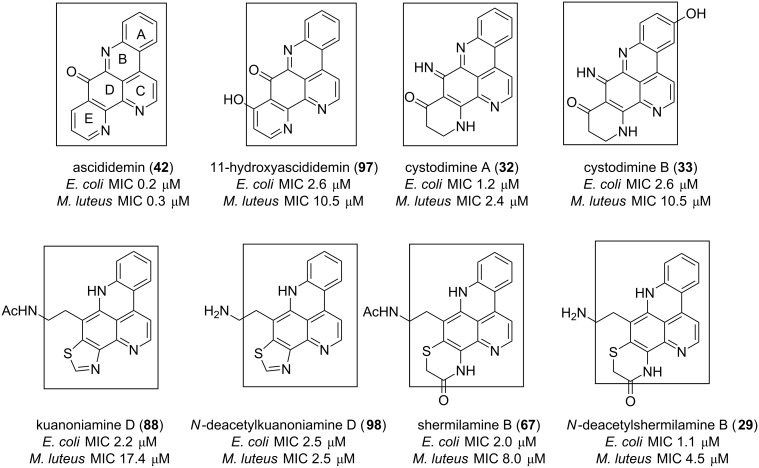
9*H*-Quinolino[4,3,2-*de*][1,10]phenanthrolin-9-one as an antibacterial pharmacophore.

Considering the cytotoxicity of **42**, **89**, **95** and **96** on vero cells, the natural product has the best core for chemical transformation since its toxicity was the lowest [70]. Other pyridoacridine alkaloids, along with ascididemin isolated from different chromotypes of the ascidian *Cystodytes dellechiajei*, demonstrated good activity against the gram negative *Escherichia coli* and the gram positive *Micrococcus luteus* bacteria [56]. Interestingly, the 9*H*-quinolino[4,3,2-*de*][1,10]phenanthrolin-9-one core corresponding to the ascididemin (**42**) structure once again has proved to be a suitable antibacterial pharmacophore (Figure 17) [56].

The decrease in the antibacterial potency of 10- and 30-fold for *E. coli and M. luteus*, respectively, was observed when the E ring contains an OH group (**97**). A similar decrease in potency was observed when the same ring is a dihydropyridone and ring A contains an OH group (**33**). No further improvement in the antibacterial activity was noted when the E ring of ascididemin is replaced with a thiazole or an oxazinole ring and a side ethylamine group is attached to the aromatic D ring (compounds **29**, **67**, **88**, **98** in Figure 17).

#### Enzymatic inhibition

**Figure 18:**
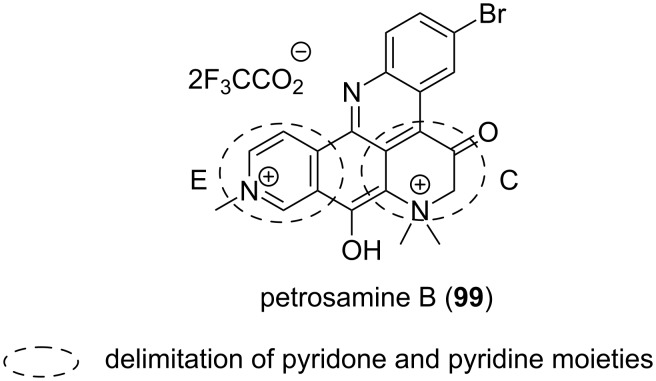
Saturated and less saturated pyridine moieties as aspartyl inhibitor cores.

Several enzymatic inhibitory activities have been described for pyridoacridine alkaloids such as meridine (**56**) that are found to exert its antifungal activity via the inhibition of nucleic acid biosynthesis [87]. Petrosamine B (**99**), isolated from the sponge *Oceanapia sp*., inhibited the *Helicobacter pylori* enzyme aspartyl semialdehyde dehydrogenase explaining it as an antibacterial effect [89]. Piperidinic phosphonates derivatives also showed the same enzyme inhibition (Figure 18) [90] and the only chemical resemblance with pyridoacridines is the piperidine ring.

Since piperidine is related to pyridine, the dihydropyridone (C) and the pyridine (E) rings in the structure of **99** could be suggested as being partly responsible for the activity.

**Figure 19:**
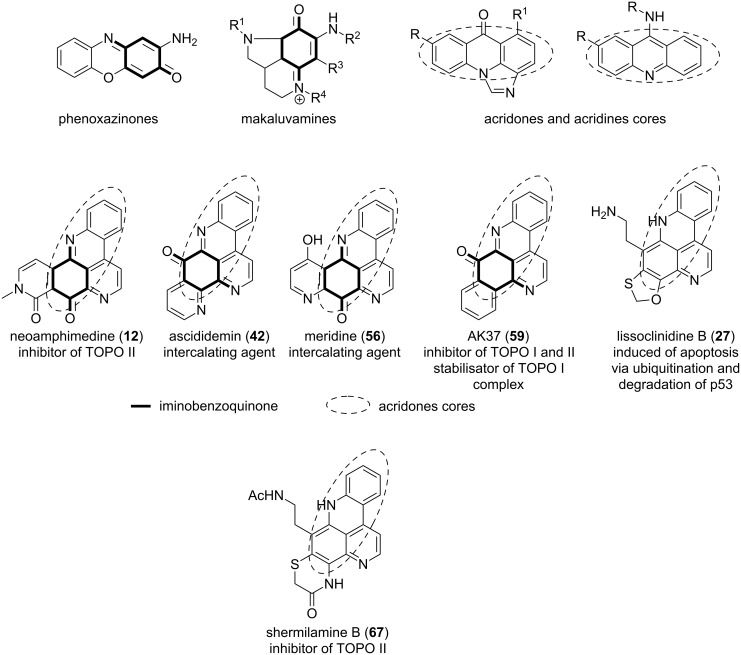
Iminobenzoquinone and acridone cores as intercalating and TOPO inhibitor motifs found in pyridoacridines alkaloids.

Furthermore, cytotoxic modes of action of pyridoacridine alkaloids include DNA-binding properties, topoisomerase (TOPO) inhibition [91] or the production of reactive oxygen species (ROS) [92–93]. It was shown that planar iminoquinone moieties and an acridine core are two pharmacophoric motifs inhibiting the proliferation of cancer cells through intercalation into DNA [42,94–95]. Compounds with such a feature can also cleave the DNA double helix or inhibit the action of TOPO [42,94–95]. These abilities have been observed in phenoxazinones [96], makaluvamines [97], acridones, and acridines alkaloids [98] (Figure 19). Ascididemin (**42**) and meridine (**56**) were found to behave like DNA intercalators and telomerase inhibitors, respectively [99]. AK37 (**59**), an ascididemin related compound was the first pyridoacridine able to stabilize the DNA-topoisomerase I complex [100] (Figure 19). Moreover, neoamphimedine (**12**) inhibits the DNA-TOPO IIα with IC_50_ at 2.0 μM [101]. It was further observed that most cytotoxic pyridoacridines acting as intercalators and inhibitors of TOPO contain both iminoquinone and acridone chemical features. Those containing only one of the motifs such as shermilamine B (**67**) and lissoclinidine B (**27**) inhibit TOPO II [42,83] or induce apoptosis via other mechanisms like ubiquitination and degradation of p53 by acting on ubiquitin ligase [44].

## Conclusion

This review compiles up-to-date information on recently identified pyridoacridines. It also describes the change in carbon shifts associated with different cores of these alkaloids and demonstrates how the carbon shift of the *N*-methyl group could be indicative of a salt form of the alkaloid. In addition, observations have been made on the change in carbon shifts of the A ring when the B ring is not aromatic. The compilation of this NMR data could be used as a library for a database prediction and could also save time with respect to structure elucidation of related natural congeners. Earlier, successful synthetic figures have also been presented as well as new, reported hypotheses on pyridoacridine biosynthesis. Furthermore, the synthesis of analogues related to some of these alkaloids has also been summarized. Biological data have been summarized in this review and different pharmacophores have been highlighted. Some of these skeletons represent good candidates for the development of new pharmaceutical leads.
